# Spectroscopic Estimation of N Concentration in Wheat Organs for Assessing N Remobilization Under Different Irrigation Regimes

**DOI:** 10.3389/fpls.2021.657578

**Published:** 2021-04-09

**Authors:** Wei Li, Xiaonan Zhou, Kang Yu, Zhen Zhang, Yang Liu, Naiyue Hu, Ying Liu, Chunsheng Yao, Xiaoguang Yang, Zhimin Wang, Yinghua Zhang

**Affiliations:** ^1^College of Agronomy and Biotechnology, China Agricultural University, Beijing, China; ^2^College of Resources and Environmental Sciences, China Agricultural University, Beijing, China; ^3^Engineering Technology Research Center for Agriculture in Low Plain Areas, Cangzhou, China

**Keywords:** hyperspectral, N concentration, PLSR, plant organ reflectance, winter wheat, N remobilization efficiency

## Abstract

Nitrogen (N) remobilization is a critical process that provides substantial N to winter wheat grains for improving yield productivity. Here, the remobilization of N from anthesis to maturity in two wheat cultivars under three irrigation regimes was measured and its relationship to organ N concentration was examined. Based on spectral data of organ powder samples, partial least squares regression (PLSR) models were calibrated to estimate N concentration (*N*_mass_) and validated against laboratory-based measurements. Although spectral reflectance could accurately estimate *N*_mass_, the PLSR-based *N*_mass_-spectra predictive model was found to be organ-specific, organs at the top canopy (chaff and top three leaves) received the best predictions (*R*^2^ > 0.88). In addition, N remobilization efficiency (NRE) in the top two leaves and top third internode was highly correlated with its corresponding N concentration change (Δ*N*_mass_) with an *R*^2^ of 0.90. Δ*N*_mass_ of the top first internode (TIN1) explained 78% variation of the whole-plant NRE. This study provides a proof of concept for estimating N concentration and assessing N remobilization using hyperspectral data of individual organs, which offers a non-chemical and low-cost approach to screen germplasms for an optimal NRE in drought-resistance breeding.

## Introduction

Wheat is one of the three major cereal crops providing over 700 million tons of grain worldwide per annual (FAO, 2019). The high yield potential and grain quality in wheat are dependent on the uptake and utilization of nutrients, from which nitrogen (N) is a key composition of chlorophyll maintaining photosynthesis assimilates that determines the grain yield ultimately. N also composes the gluten protein which is used for improving the viscoelastic properties of food products (Shewry, 2009). Previous studies have demonstrated that around 60–90% of N in grains at maturity (Barbottin et al., 2005; White et al., 2016) is remobilized from vegetative organs in cereal crops. In wheat, this proportion could be as high as 95% (Kichey et al., 2007). Post-anthesis N remobilization efficiency (NRE) is taken as an essential criterion for evaluating N recycling (Have et al., 2017). In addition to the genetic variation (Gaju et al., 2014), NRE varies between wheat organs, where leaves have higher NRE than stems and chaff (Pask et al., 2012). Besides, water deficit has been found to affect the N remobilization (Xu et al., 2006), and can result in an improved NRE in wheat (Bahrani et al., 2011). It has also been reported in rice that moderate soil drying is beneficial to improve NRE without sacrifice yield potential (Wang et al., 2016), while in most cases, water deficit or less irrigation is found to lead to lower productivity (Sun et al., 2006). Hence, balancing grain yield loss and N remobilization improvement is an eternal topic of sustainable agriculture.

N content varies significantly in different growth stages, especially after anthesis when N remobilizes from vegetative organs (including leaves, stems, and sheaths) to grains during the process of plant senescence and grain development in cereal crops (Bidinger et al., 1977; Yang and Zhang, 2006). Thus, optimizing NRE is critical to improve grain yield and N use efficiency. Typically, NRE is determined by N remobilization amount (NRA) in vegetative organs at anthesis and maturity (Gaju et al., 2014), while the estimation of NRE involves two key steps, dry mass (DM) and N concentration determination. The Kjedahl method is a traditional wet-chemical approach for measuring N concentration in plant tissues (Bertheloot et al., 2008). Alternatively, the combustion-based approach such as the Dumas method can also measure N concentration accurately (Simonne et al., 1994). However, these methods are usually labor-intensive and may cause environmental contamination (Galvez-Sola et al., 2015). Among others, the complex procedures of these laboratory-based N concentration determination methods are the major limiting factors of determining NRE in a large number of samples. Therefore, efficient evaluation of NRE requires a rapid and environmental-friendly method.

Near-infrared spectroscopy (NIRS) is considered as a “green” analytical tool for determining N concentration (Galvez-Sola et al., 2015; Gredilla et al., 2016). In addition to NIRS, the spectrum at the visible (VIS) region associated with chlorophylls absorption can also reflect nitrogen variations (Asner and Martin, 2008; Meacham-Hensold et al., 2020). Recently, spectral reflectance acquired by hyperspectral sensor (VIS and NIRS) instruments has been increasingly used for predicting N concentration in leaves (Ely et al., 2019; Meacham-Hensold et al., 2020), shoots (Nguyen et al., 2019), grains (Caporaso et al., 2018a), and the entire plants (Li et al., 2010; He J. et al., 2020). By analyzing the full-spectrum data with chemometric modeling techniques such as partial least square regression (PLSR), nutrient elements (e.g., N and micronutrients) could be estimated from hyperspectral reflectance (Vigneau et al., 2011; Serbin et al., 2012). The capability of estimating N concentration from spectroscopy has been verified to be robust in many previous studies, such as the NIRS (e.g., 830–2600 nm) for citrus leaf N (Galvez-Sola et al., 2015), the VIS-near-infrared (VIS-NIR, 400–1000 nm) spectroscopic analysis for cacao tree leaf N (Malmir et al., 2019), and the hyperspectral (VIS-NIR-SWIR, 350–2500 nm) determination for leaf N in various crop species. Despite its success in evaluating N status at leaf or canopy levels (Yu et al., 2013; Ely et al., 2019; Hasituya et al., 2020), the capability of using plant spectroscopy to characterize the N variations simultaneously in morphologically distinct organs and the feasibility of such an approach in evaluating the reallocation of N between organ tissues are rarely investigated (Vilmus et al., 2014).

As an alternative to NIRS, hyperspectral imaging (HSI) has several advantages to obtain spectral reflectance and analyze chemical properties (Caporaso et al., 2018a; Meacham-Hensold et al., 2020). HSI holds three-dimensional data that involves not only spectral data but also spatial information of the samples from the captured images (Gao and Smith, 2015; Fu et al., 2020), which makes it possible to investigate the variability of samples with texture differences. Hyperspectral images could be rapidly acquired with an HSI system under the controlled light condition as well as outdoor platforms with sunlight calibration in the field (Caporaso et al., 2018b; Malmir et al., 2019; Fu et al., 2020; Meacham-Hensold et al., 2020). Thanks to the flexibility of imaging a variety of samples, HSI enables rapid and repeatable measurements of N traits on the individual organs of the same plants, which brings new opportunities to study the variations and dynamics of plant-organ N traits and gain insights into the response of plant N reallocation to drought stress (Bertheloot et al., 2008; Hawkesford, 2017).

Therefore, this study intended to investigate the feasibility of using HSI-based spectroscopy to predict N variations in organs and to further evaluate the post-anthesis N remobilization in wheat organs under different irrigation regimes. For this aim, our main objectives were (1) to compare NRE between organs under different irrigation regimes, (2) to develop organ-specific N concentration prediction models using spectral reflectance, and (3) to evaluate N remobilization through N concentration change.

## Materials and Methods

### Field Treatments and Experimental Design

Two winter wheat (*Triticum aestivum* L.) cultivars of Jimai22 (JM22, high-yielding and cold-resistant cultivar) and Nongda399 (ND399, fast-growing and drought-resistant cultivar) were planted at Wuqiao Experimental Station of China Agricultural University, Cangzhou (37°41′N, 116°36′E), Hebei Province, China, in 2018–2019 winter wheat growing season. Soil fertility (0–20 cm) and characteristics of climate in the experimental filed are shown in Supplementary Table 1 and Supplementary Figure 1. During wheat growing season in this study, the experimental site received a total 66.6 mm precipitation, 3843 MJ/m^2^ solar radiation, 2021 sunshine hours, a daily average air temperature of 10.2°C, and a cumulative temperature above 0°C of 2977 degree-days (°C⋅d). Fertilizers were applied before sowing with a total of 240 kg N ha^–1^, 140 kg P_2_O_5_ ha^–1^, and 120 kg K_2_O ha^–1^, which were broadcast incorporated into the 20 cm surface layer of soil just before rotary tillage. Soil water content of 0–200 cm was determined and irrigated to 85% field capacity before sowing (Sun et al., 2019). After sowing, irrigations were applied at two critical crop developmental stages: upstanding (Z30) and anthesis (Z61), which are determined by using the Zadoks scale (Zadoks et al., 1974). Three irrigation regimes were as follows: W0, no irrigation after sowing; W1, irrigation (75 mm) at upstanding; and W2 (75 mm × 2), irrigation at stages of upstanding and anthesis. Each treatment includes three replicates, with nine experimental plots in total. The plot size was 10 m × 5 m, allowing for growing 30 rows of winter wheat at a row spacing of 0.15 m, with 30,000 seeds sown per plot.

### Samples Collection

**FIGURE 1:**
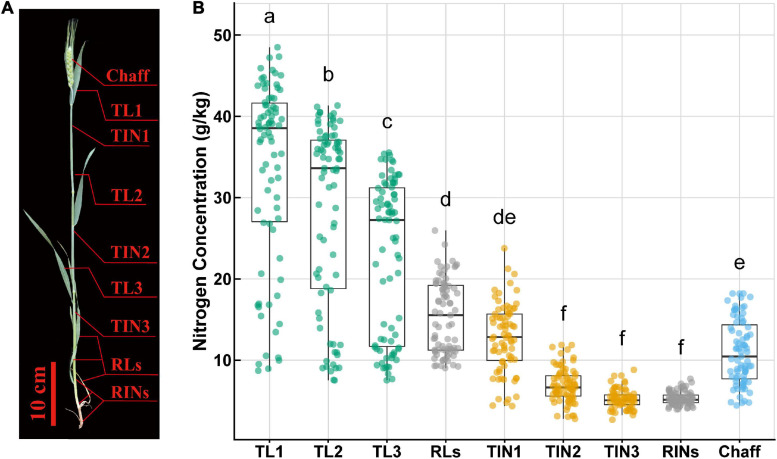
Schematic of wheat plant organs **(A)** and nitrogen concentration (*N*_mass_) variation **(B)** within and among these organs. The different lowercase letters above the boxplot show significant different mean value (*p* < 0.05). TL1, top first leaf; TL2, top second leaf; TL3, top third leaf; RLs, remaining leaves; TIN1, top first internode; TIN2, top second internode; TIN3, top third internode; RINs, remaining internodes.

The above-ground part of wheat plants from two 1 m inner rows was sampled every 5 days from flowering time (anthesis, Z31) until maturity. After field sampling, the plants were then separated into leaves, internodes (including sheaths), and chaff (spike without grain). After separated into nine parts, these samples were dried at 105°C for half an hour and then at 70°C until constant weight, and the DM was determined. As Figure 1A shows, leaf organs include TL1 (top first leaf or flag leaf laminae), TL2 (top second leaf), TL3 (top third leaf), and RLs (remaining leaves), and internode organs include TIN1 (top first internode or peduncle, including leaf sheath), TIN2 (top second internode), TIN3 (top third internode), RINs (remaining internodes), and chaff (including glume, palea, lemma, rachis, and awn) (Barraclough et al., 2014).

### Measurements of N-Related Traits

The N concentration (*N*_mass_), denoted as N mass per unit DM, was determined by using an automatic azotometer (Kjeltec 8400; Foss, Denmark) according to the Kjeldahl method (Horneck and Miller, 1998) following the manufacturer’s instructions. Post-anthesis NRE was calculated as the proportion of N in the crop component at anthesis which is not present in the vegetative parts at maturity (Gaju et al., 2014):

(1) $$NAA\left(kgNha^{-1}\right)=N_{\text{mass}}\times DM$$

(2) $$NRA\left(kgNha^{-1}\right)=NAA_{\text{Anthesis}}-NAA_{\text{Maturity}}$$

(3) $$NRE\left(\%\right)=\frac{NRA}{NAA_{\text{Anthesis}}}$$

Where NRE is the N remobilization efficiency and NRA is the amount of N remobilized (kg N ha^–1^). NAA_Anthesis_ is the amount of N (kg N ha^–1^) in vegetative organs at anthesis, and NAA_Maturity_ is the amount of N reserved in the plant at maturity. In Eq. 1, *N*_mass_ represents N mass per unit DM (g N kg^–1^), and DM represents dry mass (kg⋅ha^–1^), which was determined by the dry weight of each sample.

The N concentration change (Δ*N*_mass_) between anthesis and maturity was calculated as follows:

(4) $$\Delta N_{mass}=\frac{N_{\text{Anthesis}}-N_{\text{Maturity}}}{N_{\text{Anthesis}}}$$

Where *N*_Anthesis_ and *N*_Maturity_ represent *N*_mass_ of samples at anthesis and maturity, respectively.

The Δ*N*_mass_, N accumulation, and corresponding NRE of each organ were calculated separately. While the whole plant N accumulation is calculated as the sum of all separated organs’ accumulated N, the whole-plant NRE was then determined.

### Spectral Reflectance Acquisition

A pipeline was developed to acquire spectral reflectance from a hyperspectral imager (Supplementary Figure 2). After collecting plants from the field, separated wheat organ samples were dried and grounded to fine powders. Then these powder samples (around 3–5 g, ∼1 cm depth for each) were placed on a plate and photographed in a hyperspectral image acquisition system (Pan et al., 2019). The images were collected by an SCO710-VP hyperspectral imager (SOC, San Diego, CA, United States) covering the VIS and NIR spectra between 375 and 1050 nm at 5 nm increments for a total of 128 bands. Raw images were calibrated by the gray reference panel with known reflectance. Spectral reflectance of each sample was acquired following the procedures described in our previous work (Hu et al., 2020), by using Spectral Radiance Analysis Software (Surface Optics Corporation, United States).

### PLSR Predictive Model to Estimate *N*_mass_ From Spectra

Partial least squares regression (PLSR), which was proved to be an effective technique for building predictive models with spectral data (Serbin et al., 2012; Ely et al., 2019), was applied to develop *N*_mass_ spectra relationships. The predictive *N*_mass_ spectra model was built based on all the raw wavelength spectra for individual organs and across organs and cultivars. PLSR model was employed to predict *N*_mass_ from reflectance data with the “pls” package (Mevik et al., 2011) under the R software environment. During the procedure of model training and parameter fitting, 10 times repeated five-fold cross-validation method (Ali et al., 2017; Malmir et al., 2019) was conducted in the “caret” package (Kuhn, 2015; Heckmann et al., 2017). The optimal number of latent variables (also called model components) was determined based on the minimum predicted residual error sum of square (PRESS) statistic of the training model. Models were built on 75% of randomly selected experimental data for calibration and were used to predict the remaining (validation dataset of) 25%. A 10 times fivefold cross-validation was used to train the model. The accuracy of each model was evaluated based on the coefficients of determination (*R*^2^) and root mean square of error (RMSE) for predicted versus measured N in calibration and validation dataset. Bias was determined by the difference between the observed mean values and the predicted mean values for the validation dataset samples. Regression bias was calculated from the regression intercept. Variable importance of projection (VIP) values (Wold, 1995) of each PLSR model was evaluated to assess the relative contributions of different wavelengths over the full spectrum (Yendrek et al., 2017). VIP scores were used to identify the relative significant reflectance spectrum for each organ *N*_mass_-spectra model. In addition, wavelengths of high VIP values (>0.8) were selected to recalibrate the *N*_mass_-spectra model and compare to the all-wavelength model.

### Statistical Analysis

The actual *N*_mass_ of different time points was pooled together for all statistical analysis. Tukey HSD test was performed to compare differences between multiple groups. Linear regression analysis was conducted to study the relationship between whole-plant NRE and organ NRE. The relationships between NRE (whole plant or separate organ) and Δ*N*_mass_ were also investigated.

## Results

### N Concentration and Spectral Reflectance of the Nine Wheat Organs Powder

Chemical analysis in the laboratory showed that N concentration (*N*_mass_) varied within and among different organs across all sampling time points (Figure 1B). *N*_mass_ ranged from 2.68 to 48.5 g/kg, showing a maximum difference of 45.82 g/kg (18-fold difference). As for different organs, the flag leaf or top first leaf (TL1) showed a larger range (8.72–48.5 g/kg) and a significantly higher *N*_mass_ than the other organs. *N*_mass_ was observed with significant difference between leaf organs, while within internodes only TIN1 was significantly higher than the other internodes. Leaves showed a higher average value and wider range than internodes and chaff. Besides, in leaves or internodes, *N*_mass_ showed a vertical distribution pattern, where the top ones had a higher average value and wider range than the basal ones.

**FIGURE 2:**
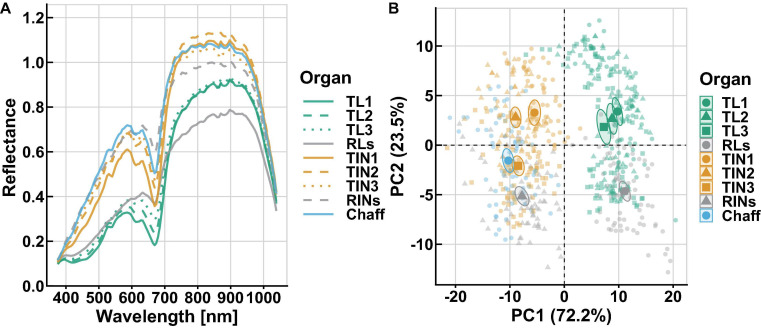
Spectra diversity within and among wheat organs. **(A)** Mean spectral reflectance for each organ is shown. **(B)** PCA plots from spectral reflectance categorized by organ group. In PCA plots, the first two PC which explained 95.7% variation, different colors and shapes correspond to each organ category, data points of individual are represented by transparent color points, and confidence ellipse and mean point of each group are shown by solid colors.

The spectral reflectance varied substantially within and among these organs’ powder (Figure 2A and Supplementary Figure 3). Across all wavelengths, an obvious peak was observed in the VIS region, while a large continuous variation in reflectance from 780 to 950 nm was detected in the NIR. Among organs, the highest reflectance was found for the chaff and top second internodes (TIN2), respectively, in the VIS and NIR regions. In contrast, the lowest was found for the flag leaves (TL1) in the VIS, as well as for the RLs in the NIR. The RINs had higher reflectance in the red (620–650 nm) and red-edge regions than other organs. It is worth noting that, in the green (505–570 nm) region, the young or green organs showed an obvious peak, while the aging organs (RLs and RINs) did not (Supplementary Figure 3). In order to understand the diversity and general properties of all samples’ spectral reflectance, principal component analysis (PCA) was applied and results showed that the first three principal components explained 98% of the variance in this set of raw spectra. PCA plots from spectral reflectance showed that organs could be divided into two subgroups, leaf and non-leaf organs, as indicated by the vertical dashed line in the figure (Figure 2B). This is consistent with the spectra variation in the VIS region, where the four leaf organs had lower reflectance than the non-leaf organs.

### N Concentration Prediction From Spectral Reflectance at Organ Level

**FIGURE 3:**
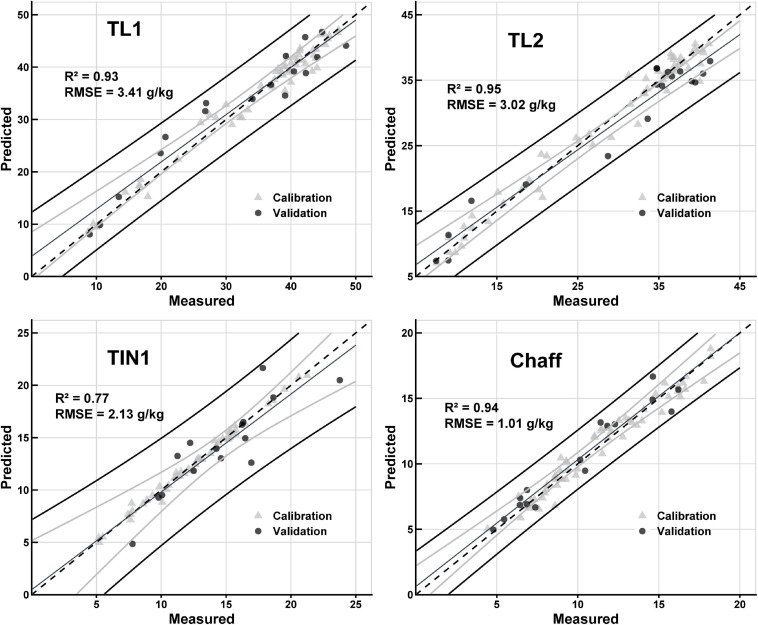
Results of PLSR predictive models for nitrogen concentration (*N*_mass_). Comparison between measured *N*_mass_ and predicted *N*_mass_ for organs of TL1, TL2, TIN1, and Chaff. In the figures, calibration (gray triangle) and validation (solid circle) data points are illustrated. The dashed line shows 1:1 line, the fine black line shows regression line, the black lines show 95% prediction interval, and gray lines show 95% confidence interval of the validation dataset. Statistic parameters (*R*^2^ and RMSE) for the validation datasets are shown in the plots.

The *N*_mass_ prediction model was built with full range wavelength spectra for individual organs and across organs (mixed organs together). Except for the RINs, all organ-specific models were able to predict *N*_mass_ (Figure 3 and Table 1). For the calibration, *R*^2^ values ranged from 0.47 to 0.97 with the calibration RMSEs varying from 0.67 to 2.80 g/kg. Surprisingly, the validation accuracy was found to be comparable to the calibration with *R*^2^ values ranged from 0.26 to 0.95 g/kg. The best organ-specific models were found for TL1, TL2, TL3, chaff, and across organs (*R*^2^ > 0.88, Table 1). The moderate prediction models were found for RLs (*R*^2^ = 0.69, RMSE = 3.00 g/kg), TIN1 (*R*^2^ = 0.77, RMSE = 2.13 g/kg), TIN2 (*R*^2^ = 0.76, RMSE = 0.97 g/kg), and TIN3 (*R*^2^ = 0.69, RMSE = 0.71 g/kg) (Table 1). In contrast, the model failed to predict *N*_mass_ in RINs (*R*^2^ = 0.26, RMSE = 0.66 g/kg). When using the selected wavelengths to model the calibration, it did not perform better than all wavelengths model (Supplementary Table 4).

**TABLE 1:** Statistics of PLSR models for *N*_mass_ of each organ and across organs.

**Dataset**		**Model components**	**Calibration**			**Validation**					
			***n***	***R*^2^**	**RMSE (g/kg)**	***n***	***R*^2^**	**RMSE (g/kg)**	**RPD**	**Bias (g/kg)**	**Regression bias (g/kg)**
	TL1	8	17	0.93	2.25	57	0.93	3.41	3.78	−0.76	3.86
	TL2	10	18	0.96	2.48	57	0.95	3.02	4.01	1.15	2.38
	TL3	3	17	0.94	2.80	55	0.94	2.51	4.11	−0.51	1.67
	RLs	3	18	0.67	2.63	59	0.69	3.00	1.60	1.29	5.79
Organ	TIN1	19	15	0.89	1.35	49	0.77	2.13	1.94	0.48	0.47
	TIN2	13	14	0.78	0.87	49	0.76	0.97	2.02	0.25	1.87
	TIN3	12	15	0.68	0.67	52	0.69	0.71	1.67	−0.17	0.84
	RINs	12	15	0.47	0.71	55	0.26	0.66	0.94	0.27	2.34
	Chaff	10	16	0.95	0.92	55	0.94	1.01	3.82	−0.32	0.62
Across organs		43	159	0.95	2.79	474	0.95	2.80	4.36	0.05	0.14

*Models were built on 75% of experimental data for calibration and used to predict the remaining (validation dataset of) 25%. Model Components is the number of components used in the predictive partial least square regression (PLSR) model. *n* is the number of samples used for modeling. RPD is the ratio of prediction to deviation. Bias is the difference between the mean observe value and the mean predicted value for the validation dataset samples. Regression bias is the regression intercept.*

### N Remobilization Varies Between Irrigation Regimes

**FIGURE 4:**
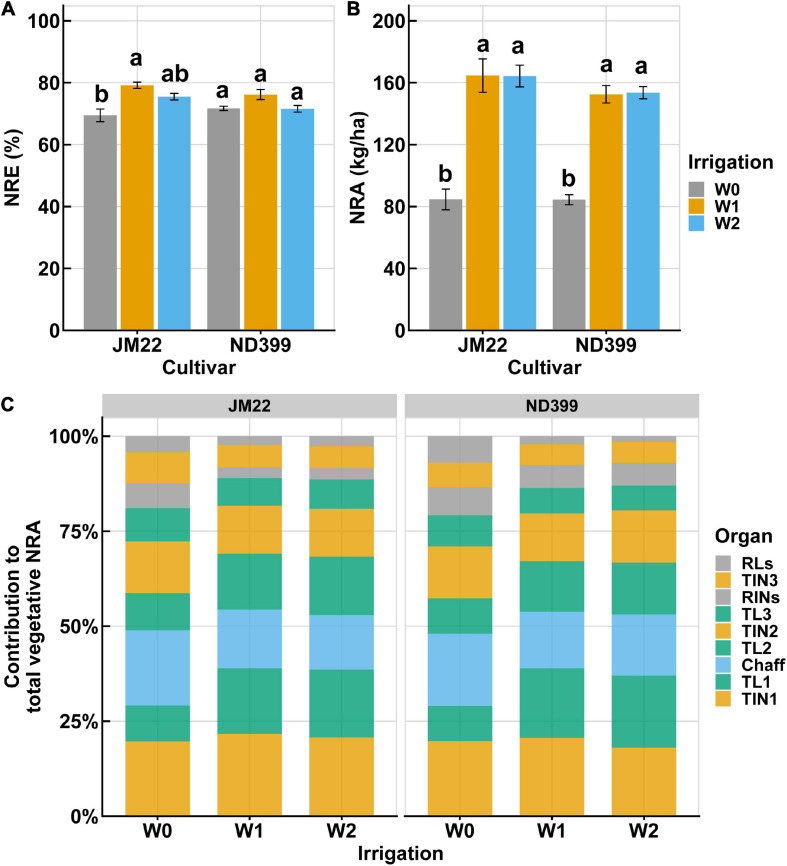
Nitrogen remobilization varies between three irrigation treatments. **(A,B)** Nitrogen remobilization efficiency (NRE) and nitrogen remobilization amount (NRA) of vegetative organs in winter wheat cultivar JM22 and ND399. Irrigation treatments are shown with three different colors. **(C)** Contribution of separated organs to total NRA.

During the anthesis and early grain developmental stages of winter wheat growing season, wheat experienced a non-precipitation period of 38 continuous days (Supplementary Figure 1b). Only a few rainfall days were recorded in the vegetative growth stages. The applied irrigation at these two critical growth stages resulted in a large difference between treatments. NRE of the two cultivars, JM22 and ND399, showed a small variation and shared a similar trend. Results showed that a low amount of irrigation (W0/W1) did not reduce NRE, compared to the higher irrigation (Figure 4A). NRA did not differ between W1 and W2, while they were much higher than W0, with a nearly doubled NRA value of W0 (Figure 4B). Figure 4C shows the contribution of nine organs to total NRA under three irrigation treatments. It is worth noting that NRA contribution from the main leaves (e.g., TL1 and TL2) decreased under W0 treatment when compared with W1 and W2 (Figure 4C). On the contrary, NRA contribution in RLs, RINs, and chaff showed increases under W0 treatment. In other organs, however, irrigation did not yield changes in NRA. Among these organs, the first two internodes, the first two leaves, and chaff are the major contribution organs, which contribute up to 80% to the total NRA (Figure 4C).

### Relationships Between N Remobilization-Related Traits

**FIGURE 5:**
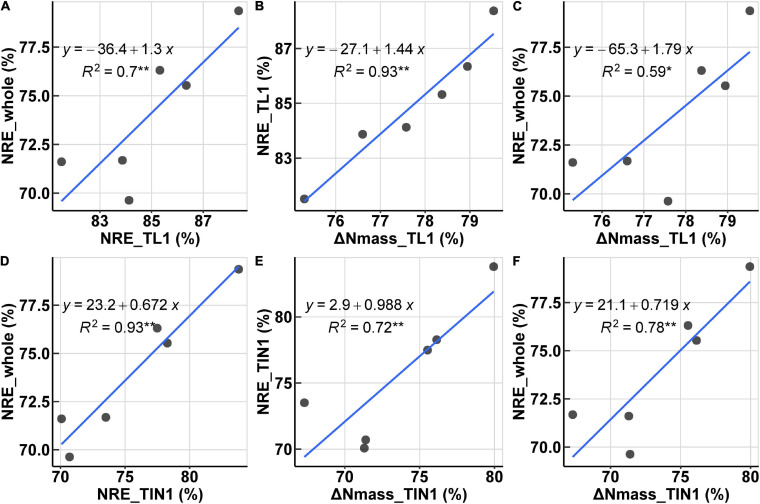
Relationships of N remobilization-related traits. **(A,D)** Relationships between NRE of separated organs and the whole plant. **(B,E)** Relationships between nitrogen concentration change (Δ*N*_mass_) and organ NRE. **(C,F)** Relationships between organ Δ*N*_mass_ and whole-plant NRE. **(A–C)** are for TL1 and **(D–F)** are for TIN1.

The organ-specific NRE was all significantly correlated with its corresponding post-anthesis Δ*N*_*mass*,_ for all organs except chaff (Table 2). For organs of TL1, TL2, and TIN3, the organ-specific NRE shared high collinearity with Δ*N*_mass_, and the best correlation was observed for TIN3, with an *R*^2^ of 0.97. Furthermore, organ-specific NRE of TL1 and TIN1 could explain 70 and 93% variation of the whole-plant NRE at significant level (*P* < 0.05) (Figure 5), while organ-specific NRE of RLs and RINs could hardly explain any variation of whole-plant NRE. In addition, the observed Δ*N*_mass_ of TIN1 could explain 78% variation of whole-plant NRE. Based on these results, it was decided to use organ-specific N concentration (e.g., TIN1) at anthesis and maturity to evaluate post-anthesis N remobilization.

**TABLE 2:** The coefficients of determination (*R*^2^) between whole plant and organ NRE, between organ NRE and organ Δ*N*_mass_, and between whole-plant NRE and organ Δ*N*_mass_ among wheat organs.

	**NRE_whole**		**NRE_organ**		**NRE_whole**	
	**∼ NRE_organ**		**∼Δ*N*_mass_**		**∼Δ*N*_mass_**	
**Organ**	***R*^2^**	***p*-value**	***R*^2^**	***p*-value**	***R*^2^**	***p*-value**
TL1	**0.7**	**0.039**	0.93	0.0016	0.59	0.073
TL2	0.44	0.15	0.79	0.017	0.08	0.58
TL3	0.08	0.59	0.93	0.0017	0	0.95
RLs	0	0.96	0.68	0.044	0.01	0.83
TIN1	**0.93**	**0.002**	0.72	0.034	**0.78**	**0.02**
TIN2	0.52	0.11	0.84	0.0104	0.64	0.058
TIN3	0.67	0.046	0.97	0.0004	0.66	0.051
RINs	0.02	0.79	0.78	0.019	0.04	0.71
Chaff	0.5	0.12	0.51	0.11	0.05	0.69

*The values in bold indicate coefficients with better performance at significant level (*p* < 0.05).*

## Discussion

### NRE Assessment Through Spectral Reflectance Estimated *N*_mass_

Winter wheat production has been improved with high-input cropping systems over the past decades, so does the adaptive capacity of cultivars to less optimal water availability conditions (Voss-Fels et al., 2019). However, our understanding on the NRE adaptivity to drought is still limited due to the lack of efficient, repeatable approaches for evaluating the NRE. In this study, NRE variations under contrast irrigation regimes were evaluated by using hyperspectral-based N measures. Results revealed that under low precipitation, mild/moderate irrigation (W1) improved NRE a bit compared to the higher irrigation (W2), while whether W0 leads to a low NRE was dependent on the cultivar (Figure 4A). These results suggest that limited irrigation, which is beneficial to save water resources (Yang and Zhang, 2006; Li et al., 2019), is a cost-efficient approach for improving N use efficiency. On the other hand, grain yield was generally found to be decreased when mild irrigation was applied (Zaveri and Lobell, 2019), which is consist with our results that low yield was received from low irrigations (Supplementary Table 2). Balancing the grain yield and NRE improvement when applying water-saving management is always a trade-off. Although several studies reported that drying condition could benefit grain yield and nutrient use efficiency simultaneously (Wang et al., 2016; He G. et al., 2020), it is still challenging to determine the extent to which the irrigation should be optimized (Nguyen and Kant, 2018; Prey and Schmidhalter, 2019).

Our results not only confirmed the power of hyperspectral sensors in precisely estimating N concentration (Table 1) but also demonstrated the potential of assessing NRE variations through spectral reflectance (Table 2 and Figure 5). By predicting N concentration, N concentration change (Δ*N*_mass_) between the anthesis and post-anthesis (Barbottin et al., 2005; Kong et al., 2016) was able to be further estimated. The observed correlation between the actually measured NRE and Δ*N*_mass_ (Table 2) suggested the possibility of evaluating N remobilization directly through spectral measurements. Our results have added to the growing evidence that plant spectroscopy could facilitate the study of N remobilization in response to irrigation (or fertilization) management that affects N remobilization, especially in terms of the timing and duration at organ level (Nehe et al., 2020). Notably, NRE measurement is a time-consuming and costly process which involves measurement of *N*_mass_ and dry matter simultaneously at the two critical stages (anthesis and maturity). Additionally, the whole-plant NRE could be estimated by acquiring spectral data of TIN1 powder samples at anthesis and maturity, suggesting a promising application in selecting N-efficient cultivars (Nguyen and Kant, 2018). For NRE correlations among organs, considering post NRE was determined by the contribution of organs. However, cultivars and environmental variations may have an influence on the relationships between NRE and Δ*N*_mass_, and also affect the use of specific organ data in assessing the whole-plant NRE. Further investigations are needed to improve the understanding of correlations between NRE and Δ*N*_mass_. Our study only provides a proof of concept for evaluating N remobilization under controlled light conditions following simple sample preparations (spectra acquired from organ powders). Nevertheless, it is still challenging to perform organ-specific HSI measurements in the field. Future improvement of the proposed method should be investigated by measuring individual plants and organs directly in the field.

### Organ-Level Predictive Models

Predictive models calibrated across organs did not always succeed in predicting individual organs (data not shown). Similar results were also reported in models using leaf spectral reflectance in diverse species (Heckmann et al., 2017; Yendrek et al., 2017). This difference was validated by performing PCA for the spectral reflectance, in which leaf and non-leaf organs are statistically different as tested by a permutational multivariate analysis of variance (Anderson, 2017). These differences between leaf and non-leaf destructive organs might be attributed to their divergent biochemical composition. PCA results suggest that, even within the same type of organ (e.g., leaf and internode groups), large variations exist. Here, N concentrations of different vegetative organs were investigated and results suggest considerable variations distributed vertically among these organs (Figure 1B). These findings are similar to previous N partitioning research in wheat (Barraclough et al., 2014; Gaju et al., 2014). Collectively, our results confirmed the usefulness of considering organ-specific models.

In this study, similarly high predictive power (*R*^2^ ∼ 0.90) (Table 1) was achieved for leaf organs compared to previous *N*_mass_ estimations at leaf level (Serbin et al., 2012; Yendrek et al., 2017; Coast et al., 2019; Ely et al., 2019). Surprisingly, prediction for the chaff organ was comparable (*R*^2^ = 0.94) to the top leaf organs, which might be related to the specific role of chaff in maintaining a high N accumulation by acting as a temporary sink and source for N (Kong et al., 2016). Also, senescent leaves and internodes achieved relatively low predictive power (Table 1), which was similar to vertical canopy N predictions in rice (He J. et al., 2020). Small variations in spectra and N concentration may account for the low prediction power in these senescence organs. The relatively low N concentration might reduce the possibility of being detected by spectra, which is confirmed by a low coefficient of variation (CV) observed in the lower prediction models (Supplementary Table 5). In this situation, VIP values at some dominating wavelength were decreased (Supplementary Figure 4), resulting in a low contribution to the prediction (Yendrek et al., 2017). Regarding to the effect of organ on wavelength sore, one possible reason might be attributed from the biochemical composition between organs which more chlorophylls content is reserved in leaf or young organs dominating the reflection of N variations (Meacham-Hensold et al., 2020). For a robust PLSR model for predicting N in these organs having small variations, it might be possible by collecting a diverse range of *N*_mass_ data across more growth conditions and developmental stages or diverse genetic resources.

### Hyperspectral Imaging as a Rapid and Cost-Effective Approach to Acquire Spectral Reflectance

This study used HSI to acquire reflectance data for estimating N concentration and assessing NRE variations of dried samples. As a spectroscopic technology, the biggest advantage is that the method is cost-efficient and environmentally friendly (Gredilla et al., 2016), and thus is suitable for repeated use. Due to its flexibility and convenience, spectral reflectance for a large number of samples was able to be acquired. For example, in our experiment, 18 samples were photographed (Supplementary Figure 2) in one image, making it possible for us to acquire more than 1000 samples within 10 h. To reduce the spatial complexity of analyzing the spectra while still taking the advantage of image data, regions of interest are typically selected to acquire average spectra (Malmir et al., 2019). Despite the benefits of HSI, this study only focused on the uses of organ powder samples and the average spectra of each sample, without discriminating spatial variations in each organ. The simplified approach for evaluating the whole-plant NRE through organ part (TIN1) spectra will facilitate balancing the irrigation and N management and screening high NRE cultivars. In our study, NRE of JM22 is superior to that in ND399 under sufficient water treatments (Supplementary Figure 5, W1 and W2). Hence, it is possible to screen high NRE cultivars under specific irrigation conditions. This study provides a proof of concept for evaluating N remobilization of wheat organs and whole plant through hyperspectral reflectance data. It is anticipated that HSI will be a promising tool for analyzing NRE spatial variation and its dynamics to uncover the underlying mechanism of N remobilization.

## Conclusion

In this study, the N remobilization under three irrigation regimes was investigated using HSI. Results showed that NRE varied among irrigation regimes after sowing, where the mild irrigation (W1) achieved the best NRE, but the NRE reduced if no irrigation was applied. NRE in individual organs was correlated highly with the whole-plant NRE. Spectra-based models successfully predicted the N concentration in each organ. The N concentration change in a single organ (e.g., TIN1) between anthesis and maturity could explain 78% of the variation in the whole plant NRE. This study demonstrates the use of HSI in estimating N concentration and to aid the assessment of NRE variations from the organ to whole-plant levels, which holds great promise for guiding precision irrigation and N management for optimized NRE.

## Data Availability Statement

The original contributions presented in the study are included in the article/Supplementary Material. Further inquiries can be directed to the corresponding author/s.

## Author Contributions

WL, XZ, ZW, and YZ conceived and designed the experiments. WL, XZ, ZZ, YaL, and NH performed the experiments. WL, XZ, YiL, CY, and XY analyzed the data. WL, XZ, KY, and YZ wrote the manuscript. All authors read and approved the final manuscript.

## Conflict of Interest

The authors declare that the research was conducted in the absence of any commercial or financial relationships that could be construed as a potential conflict of interest.
