# Supplementary material for: Spectroscopic Estimation of N Concentration in Wheat Organs for Assessing N Remobilization Under Different Irrigation Regimes
Source: Front Plant Sci. 2021 Apr 9;12:657578. doi: 10.3389/fpls.2021.657578 (PMC8062884; doi:10.3389/fpls.2021.657578)
Supplement: Supplementary file 1 [file Image_1.pdf]

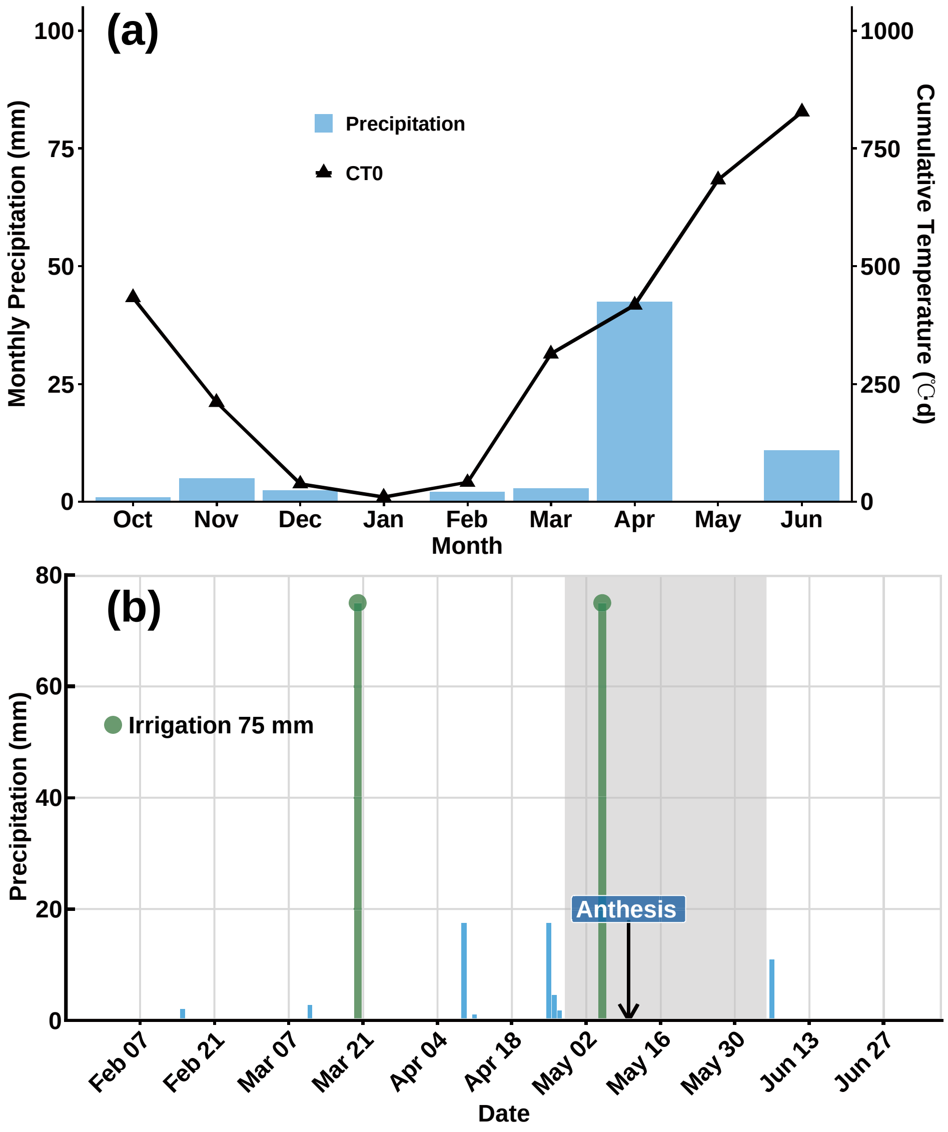

Supplementary Figure 1. Monthly weather across the wheat growing season (a) and daily precipitation in critical developmental stage (b). CT 0, cumulative temperature above 0 ℃ (degree-days, ℃·d), for 0 ℃ is the lower limit for wheat to grow. No precipitation occurred before and after anthesis, the duration lasts for 38 days (grey shadow) until harvest. Irrigation of 75 mm was applied at two critical growing stages (light green points).
