# Supplementary material for: Spectroscopic Estimation of N Concentration in Wheat Organs for Assessing N Remobilization Under Different Irrigation Regimes
Source: Front Plant Sci. 2021 Apr 9;12:657578. doi: 10.3389/fpls.2021.657578 (PMC8062884; doi:10.3389/fpls.2021.657578)
Supplement: Supplementary file 2 [file Image_2.pdf]

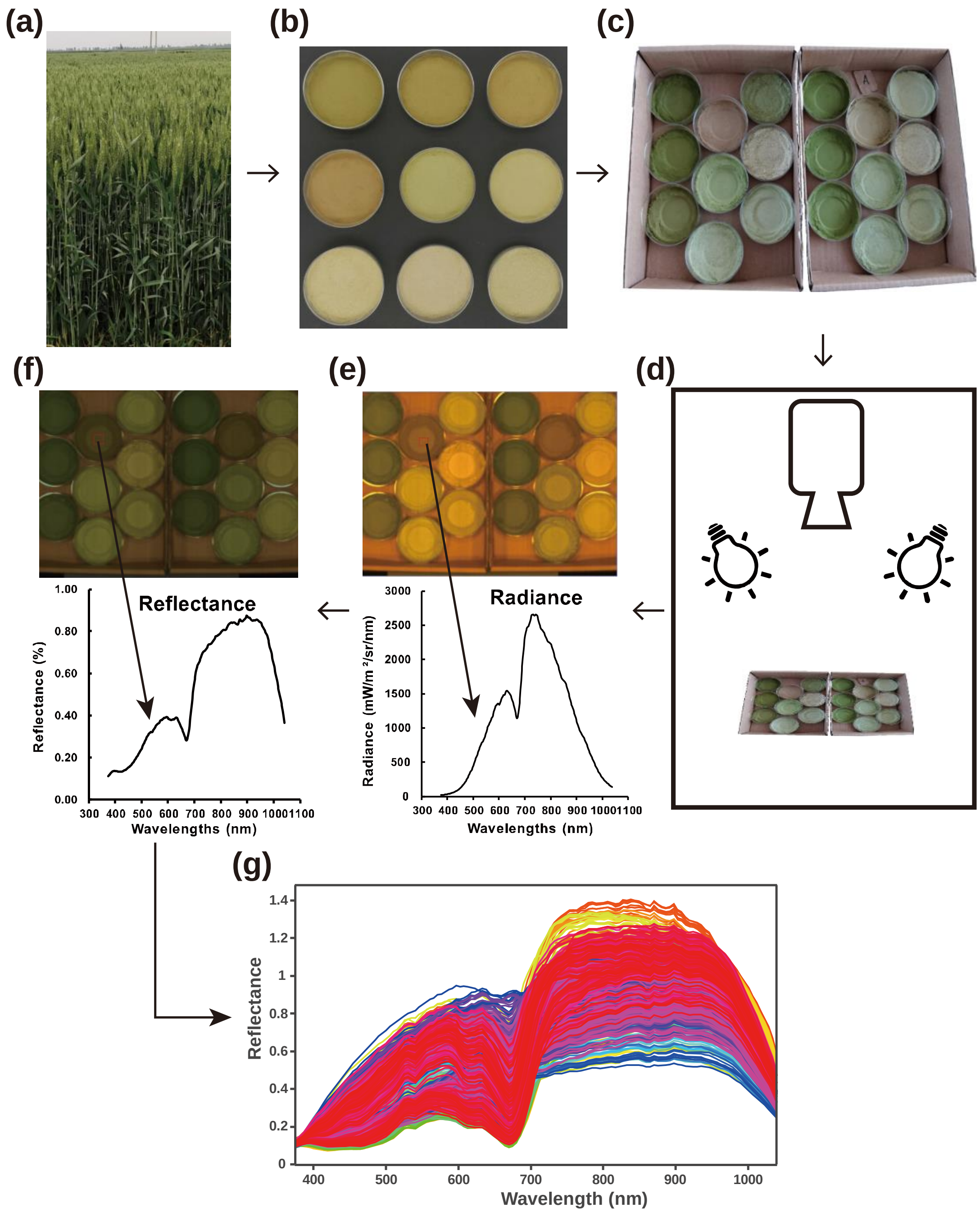

Supplementary Figure 2. Pipeline for acquiring spectral reflectance of each sample from hyperspectral image. Collect wheat plants from the field (a), and mill the separated organs to a fine powder (b). Then transfer the powder samples to a box with 18 dishes (c) and photographed in a hyperspectral imaging acquisition system (d). Raw spectral radiance image (e) were calibrated to reflectance image (f) and average spectral reflectance of each sample were extracted from the calibrated image (g). Radiance and reflectance spectra were shown in (e) and (f).
