# Supplementary material for: Spectroscopic Estimation of N Concentration in Wheat Organs for Assessing N Remobilization Under Different Irrigation Regimes
Source: Front Plant Sci. 2021 Apr 9;12:657578. doi: 10.3389/fpls.2021.657578 (PMC8062884; doi:10.3389/fpls.2021.657578)
Supplement: Supplementary file 3 [file Image_3.pdf]

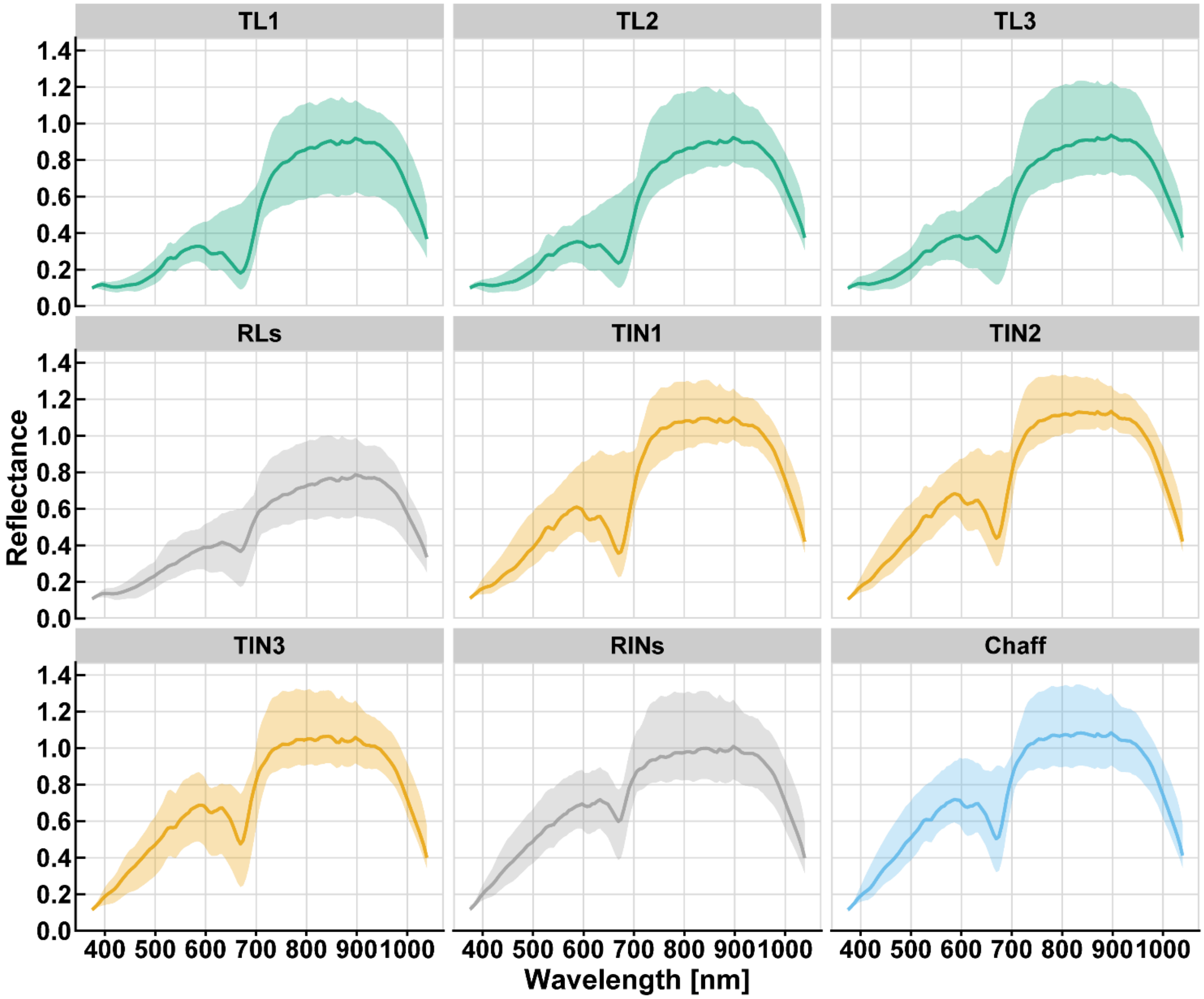

Supplementary Figure 3. Mean, minimum and maximum spectral reflectance for separated organs. Solid line represents mean reflectance, shadow under the line represents upper and lower limits of the corresponding spectral reflectance.
