# Supplementary material for: Spectroscopic Estimation of N Concentration in Wheat Organs for Assessing N Remobilization Under Different Irrigation Regimes
Source: Front Plant Sci. 2021 Apr 9;12:657578. doi: 10.3389/fpls.2021.657578 (PMC8062884; doi:10.3389/fpls.2021.657578)
Supplement: Supplementary file 4 [file Image_4.pdf]

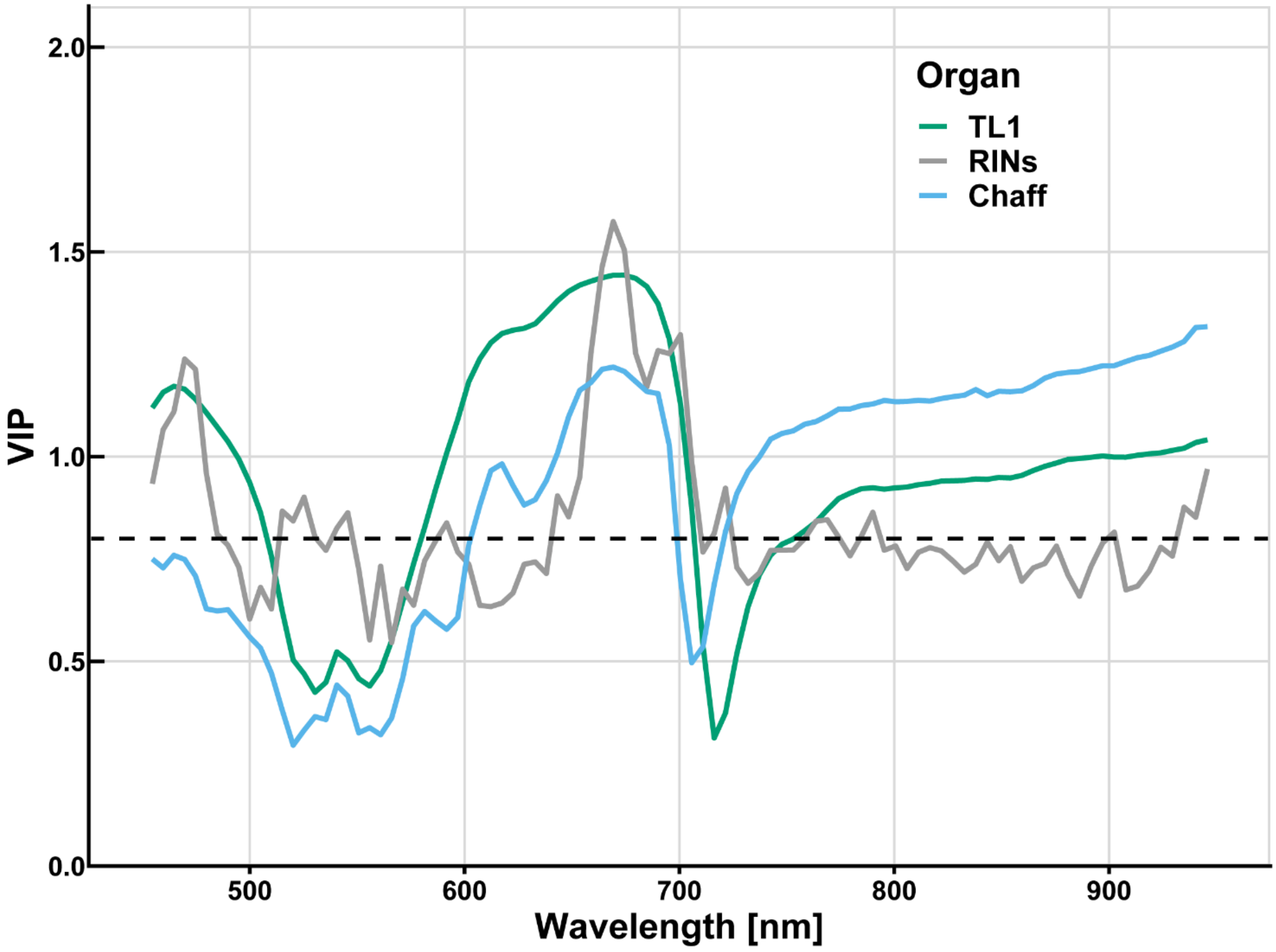

Supplementary Figure 4. Variable importance of projection (VIP) of PLSR N_{mass} predictive models for TL1, chaff and RINs presented in this study. VIP values of 0.8 (dashed line) is shown. TL1 and chaff achieved high predictive power, while RINs achieved lower predictive power.
