# Supplementary material for: Spectroscopic Estimation of N Concentration in Wheat Organs for Assessing N Remobilization Under Different Irrigation Regimes
Source: Front Plant Sci. 2021 Apr 9;12:657578. doi: 10.3389/fpls.2021.657578 (PMC8062884; doi:10.3389/fpls.2021.657578)
Supplement: Supplementary file 5 [file Image_5.pdf]

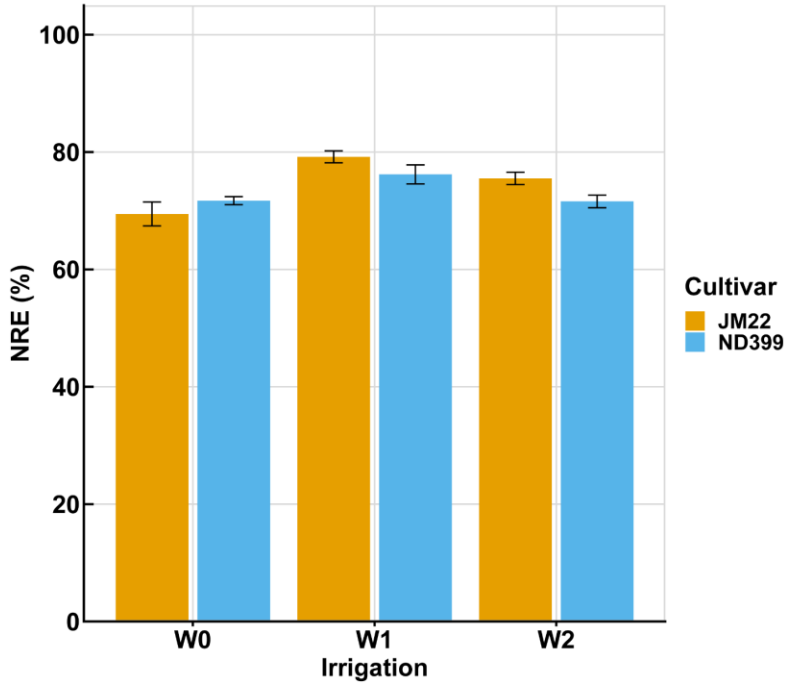

Supplementary Figure 5. Nitrogen remobilization efficiency (NRE) comparison between two cultivars for three irrigation regimes.
