# Supplementary material for: Spectroscopic Estimation of N Concentration in Wheat Organs for Assessing N Remobilization Under Different Irrigation Regimes
Source: Front Plant Sci. 2021 Apr 9;12:657578. doi: 10.3389/fpls.2021.657578 (PMC8062884; doi:10.3389/fpls.2021.657578)
Supplement: Supplementary file 6 [file Table_1.docx]

**Supplementary Table 1.** Soil fertility (0-20 cm) in wheat growth seasons of this study

|  | **Organic matter**  **(g/kg)** | **Total nitrogen**  **(g/kg)** | **Available K**  **(mg/kg)** | **Available P**  **(mg/kg)** |
| --- | --- | --- | --- | --- |
| **2018-2019** | 12.6 | 1.1 | 103.9 | 45.5 |
