# Supplementary material for: Spectroscopic Estimation of N Concentration in Wheat Organs for Assessing N Remobilization Under Different Irrigation Regimes
Source: Front Plant Sci. 2021 Apr 9;12:657578. doi: 10.3389/fpls.2021.657578 (PMC8062884; doi:10.3389/fpls.2021.657578)
Supplement: Supplementary file 7 [file Table_2.docx]

**Supplementary Table 2.** Anthesis and maturity day and grain yield for each cultivar in three irrigation regimes.

| Cultivar | Irrigation | Anthesis day | Maturity day | Grain yield (kg/hm^2^) |
| --- | --- | --- | --- | --- |
| JM22 | W0 | 2019/5/7 | 2019/5/30 | 5409 |
|  | W1 | 2019/5/8 | 2019/6/4 | 8742 |
|  | W2 | 2019/5/9 | 2019/6/8 | 9848 |
| ND399 | W0 | 2019/5/8 | 2019/5/30 | 5025 |
|  | W1 | 2019/5/8 | 2019/6/3 | 8274 |
|  | W2 | 2019/5/9 | 2019/6/8 | 9377 |
