# Supplementary material for: Spectroscopic Estimation of N Concentration in Wheat Organs for Assessing N Remobilization Under Different Irrigation Regimes
Source: Front Plant Sci. 2021 Apr 9;12:657578. doi: 10.3389/fpls.2021.657578 (PMC8062884; doi:10.3389/fpls.2021.657578)
Supplement: Supplementary file 8 [file Table_3.docx]

**Supplementary Table 3.** Cross validation results of predicting N concentration (*N*_mass_) with PLSR for each organ and across organs in two wheat cultivars, respectively.

| Organ | JM22 | | | |  | ND399 | | | |
| --- | --- | --- | --- | --- | --- | --- | --- | --- | --- |
|  | Model Component | N | R^2^ | RMSE |  | Model Component | N | R^2^ | RMSE |
| TL1 | 11 | 37 | 0.95 | 2.60 |  | 4 | 37 | 0.94 | 2.71 |
| TL2 | 13 | 38 | 0.93 | 2.88 |  | 4 | 37 | 0.95 | 2.46 |
| TL3 | 5 | 37 | 0.93 | 2.40 |  | 5 | 35 | 0.92 | 2.75 |
| RLs | 18 | 39 | 0.82 | 2.15 |  | 9 | 38 | 0.64 | 2.47 |
| TIN1 | 23 | 31 | 0.83 | 1.45 |  | 3 | 33 | 0.79 | 1.98 |
| TIN2 | 19 | 35 | 0.62 | 1.19 |  | 11 | 28 | 0.55 | 1.03 |
| TIN3 | 3 | 30 | 0.56 | 0.48 |  | 21 | 37 | 0.85 | 0.56 |
| RINs | 5 | 36 | 0.37 | 0.55 |  | 14 | 34 | 0.37 | 0.74 |
| Chaff | 12 | 36 | 0.90 | 1.20 |  | 14 | 35 | 0.88 | 1.28 |
| Across organs | 42 | 319 | 0.95 | 2.91 |  | 33 | 314 | 0.95 | 2.62 |

Models were built with ten times five-fold cross-validation on the experimental data. Model Components is the number of components used in the predictive partial least square regression (PLSR) model. N is the number of samples used for modeling.
