# Supplementary material for: Spectroscopic Estimation of N Concentration in Wheat Organs for Assessing N Remobilization Under Different Irrigation Regimes
Source: Front Plant Sci. 2021 Apr 9;12:657578. doi: 10.3389/fpls.2021.657578 (PMC8062884; doi:10.3389/fpls.2021.657578)
Supplement: Supplementary file 9 [file Table_4.docx]

**Supplementary Table 4.** Statistics of PLSR models for N concentration (*N*_mass_) of each organ and across organs with selected wavelengths.

| Dataset | | Model  Components |  | Calibration | |  |  |  | | Validation | | | |
| --- | --- | --- | --- | --- | --- | --- | --- | --- | --- | --- | --- | --- | --- |
|  |  |  | N | R^2^ | RMSE  (g/kg) |  | N | R^2^ | RMSE  (g/kg) | | RPD | Bias  (g/kg) | Regression  Bias (g/kg) |
|  | TL1 | 8 | 17 | 0.94 | 2.17 |  | 57 | 0.93 | 3.33 | | 3.87 | -0.90 | 3.06 |
|  | TL2 | 9 | 18 | 0.95 | 2.52 |  | 57 | 0.95 | 3.00 | | 4.04 | 1.19 | 2.27 |
|  | TL3 | 3 | 17 | 0.93 | 2.77 |  | 55 | 0.94 | 2.56 | | 4.03 | -0.26 | 1.74 |
|  | RLs | 5 | 18 | 0.69 | 2.61 |  | 59 | 0.69 | 2.78 | | 1.73 | -0.18 | 5.48 |
| Organ | TIN1 | 20 | 15 | 0.88 | 1.44 |  | 49 | 0.81 | 2.21 | | 1.87 | 0.15 | -1.95 |
|  | TIN2 | 12 | 14 | 0.75 | 0.87 |  | 49 | 0.74 | 0.98 | | 2.01 | 0.08 | 1.63 |
|  | TIN3 | 18 | 15 | 0.71 | 0.69 |  | 52 | 0.76 | 0.70 | | 1.70 | -0.25 | 0.17 |
|  | RINs | 14 | 15 | 0.47 | 0.24 |  | 55 | 0.24 | 0.68 | | 0.90 | 0.34 | 2.54 |
|  | Chaff | 10 | 16 | 0.92 | 1.16 |  | 55 | 0.95 | 0.93 | | 4.17 | -0.38 | 0.35 |
| Across organs | | 30 | 159 | 0.92 | 3.54 |  | 474 | 0.92 | 3.46 | | 3.53 | 0.07 | 1.11 |

Models were built on 75% of experimental data for calibration and used to predict the remaining (validation dataset of) 25%. Model Components is the number of components used in the predictive partial least square regression (PLSR) model. N is the number of samples used for modeling. RPD is the ratio of prediction to deviation. Bias is the difference between the mean observe value and the mean predicted value for the validation dataset samples. Regression bias is the regression intercept.
