# Supplementary material for: Spectroscopic Estimation of N Concentration in Wheat Organs for Assessing N Remobilization Under Different Irrigation Regimes
Source: Front Plant Sci. 2021 Apr 9;12:657578. doi: 10.3389/fpls.2021.657578 (PMC8062884; doi:10.3389/fpls.2021.657578)
Supplement: Supplementary file 10 [file Table_5.docx]

**Supplementary Table 5.** Statistical analysis for the N concentration (*N*_mass_, g/kg) of nine wheat organs.

| Organ | Mean | Min | Max | CV (%) |
| --- | --- | --- | --- | --- |
| TL1 | 33.6 | 8.72 | 48.5 | 33.9 |
| TL2 | 28.4 | 7.52 | 41.3 | 39.8 |
| TL3 | 22.8 | 7.53 | 35.5 | 41.9 |
| RLs | 15.4 | 9.04 | 26.0 | 29.2 |
| TIN1 | 12.8 | 4.36 | 23.8 | 33.0 |
| TIN2 | 6.91 | 2.79 | 11.9 | 30.6 |
| TIN3 | 5.23 | 2.68 | 8.82 | 23.0 |
| RINs | 5.30 | 3.95 | 7.72 | 15.4 |
| Chaff | 11.0 | 4.43 | 18.2 | 36.1 |

CV, coefficient of variation
